# Organ-Tumor-on-a-Chip for Chemosensitivity Assay: A Critical Review

**DOI:** 10.3390/mi7080130

**Published:** 2016-07-28

**Authors:** Navid Kashaninejad, Mohammad Reza Nikmaneshi, Hajar Moghadas, Amir Kiyoumarsi Oskouei, Milad Rismanian, Maryam Barisam, Mohammad Said Saidi, Bahar Firoozabadi

**Affiliations:** School of Mechanical Engineering, Sharif University of Technology, 11155-9567 Tehran, Iran; morenik1367@gmail.com (M.R.N.); moghadas_stu@yahoo.com (H.M.); amirkiumarsi@gmail.com (A.K.O.); milad_rismanian@yahoo.com (M.R.); barisam.m@gmail.com (M.B.); firoozabadi@sharif.edu (B.F.)

**Keywords:** tumor-on-a-chip, cancer in microfluidics, drug efficacy testing, in vitro assays, concentration gradient generators, microchip cell culture, spheroids, tumor microenvironment

## Abstract

With a mortality rate over 580,000 per year, cancer is still one of the leading causes of death worldwide. However, the emerging field of microfluidics can potentially shed light on this puzzling disease. Unique characteristics of microfluidic chips (also known as micro-total analysis system) make them excellent candidates for biological applications. The ex vivo approach of tumor-on-a-chip is becoming an indispensable part of personalized medicine and can replace in vivo animal testing as well as conventional in vitro methods. In tumor-on-a-chip, the complex three-dimensional (3D) nature of malignant tumor is co-cultured on a microfluidic chip and high throughput screening tools to evaluate the efficacy of anticancer drugs are integrated on the same chip. In this article, we critically review the cutting edge advances in this field and mainly categorize each tumor-on-a-chip work based on its primary organ. Specifically, design, fabrication and characterization of tumor microenvironment; cell culture technique; transferring mechanism of cultured cells into the microchip; concentration gradient generators for drug delivery; in vitro screening assays of drug efficacy; and pros and cons of each microfluidic platform used in the recent literature will be discussed separately for the tumor of following organs: (1) Lung; (2) Bone marrow; (3) Brain; (4) Breast; (5) Urinary system (kidney, bladder and prostate); (6) Intestine; and (7) Liver. By comparing these microchips, we intend to demonstrate the unique design considerations of each tumor-on-a-chip based on primary organ, e.g., how microfluidic platform of lung-tumor-on-a-chip may differ from liver-tumor-on-a-chip. In addition, the importance of heart–liver–intestine co-culture with microvasculature in tumor-on-a-chip devices for in vitro chemosensitivity assay will be discussed. Such system would be able to completely evaluate the absorption, distribution, metabolism, excretion and toxicity (ADMET) of anticancer drugs and more realistically recapitulate tumor in vivo-like microenvironment.

## 1. Introduction

Though described as a modern disease, cancer, originally named by Hippocrates (460–370 BC), is one of the oldest diseases of human beings as well as other animals. Edwin Smith Papyrus describes breast tumors more than 5000 years ago, with “there is no treatment” under the treatment options [1]. This disease seems to be hidden under the shadows of other fatal diseases and has been more highlighted in the last 100 years as one of the leading causes of death worldwide. Its occurrence is directly related to patient’s age and thus illustrates the underlying reason of its frequency in recent years, with over 80-year life spans. 

Throughout this paper, we concise the term “tumor” to malignant neoplasms showing six hallmarks of cancerous cells including [2]: (1) capability of cells to grow and divide without stimulus signals; (2) ignoring anti-growth signals; (3) inability to undergo apoptosis; (4) gaining immortality potential; (5) producing extra blood vessels (angiogenesis) and (6) invading tissue and spreading to other organs (metastasis).

Chemotherapy, with surgical excision, radiotherapy and immunotherapy is among the most common curing options. However, in most cases, recurrence of tumor as well as its metastasis to other tissues has made treatment ineffective.

Conventionally, in preclinical drug development, two methods of animal work and/or two-dimensional (2D) or three-dimensional (3D) laboratory cell cultures are used to evaluate the efficacy and safety of a drug candidate in vivo and in vitro, respectively. While animal study provides a systemic environment for the tumor growth, it lacks the realistic response of human body. On the other hand, static monolayer 2D and more realistic 3D laboratory cell culture plates lack the systemic nature of living cells. It highlights the importance of an alternative platform to further understanding the complex nature of this disease as well as to develop effective therapeutic agents.

Microfluidics, which is a study of fluid flow in micron-size domains, proves to be an effective technology in cancer study both in vivo and in vitro. For in vivo study, it is related to targeted drug delivery systems using smart carriers [3,4,5]. With the emerging concept of lab-on-a-chip, in vitro microfluidic devices are closely linked to tissue engineering and regenerative science promising a great step toward personalized medicine [6].

One of the most important features of a cancerous tumor, which highly affects its therapeutic response to anti-cancer drugs, is its complex microenvironment including blood vessels, fibroblasts immune cells and extracellular matrix (ECM). In addition, the above-mentioned hallmarks of malignant neoplasms and other special traits of tumor such as Enhanced Permeability and Retention Effect (EPRF) and oxygen deficiency are related to its microenvironment. Therefore, cancer cells alone cannot be used to simulate tumor behavior [7]. On the other hand, cancer is a genetic disease and each patient may react differently to a specific therapy. It signifies the importance of paradigm shift toward personalized therapy. However, one of the most challenging issues is the micron size amount of tumor that would be available from needle biopsy. It makes traditional sets up of drug testing ineffective where large amount of cells are required, for instance on conventional 96-well plates.

Capability of microfluidic devices to integrate all the necessary components in a less than 1-inch silicon chip along with the advances in micro electro mechanical systems (MEMS) led to highly efficient lab-on-a-chip devices (LOC) [8,9,10]. Other unique features of a microfluidic platform such as its perfect length scale fitting at cellular and tissue levels as well as very small amount of required agents made them an excellent choice for biological applications [11]. The collaboration between engineers, biologists and medical doctors led to the advent of organ-on-a-chip [12,13,14,15,16,17,18,19]. It can mimic all the physiological behaviors of the living organ and facilitate disease modeling for better understanding the nature of cellular changes in vitro [20].

In the case of treating cancer, it can provide excellent medium to recapitulate key elements of tumor hallmarks (microenvironment) as well as a high throughput screening tool for further investigation toward personalized medicine. Microfluidic platform seems to be the next generation of cell culture technique, which sooner or later will replace the conventional techniques in all clinics and hospitals. This platform is called tumor-on-a-chip. 

It can also be used in detecting circulating tumor cells (CTCs) in blood flow which may lead to early diagnosis of cancer [21,22]. Recent microfluidic advances in this field as well as required process to translate CTC biomarkers to clinical practice are reviewed elsewhere [23,24]. 

The capability of microfluidic tumor-on-a-chip devices has been evaluated to investigate the efficacy of targeted drug delivery of nanoparticles (NPs) [25,26]. Barua and Mitragotri [27] discussed and reviewed the advances regarding overcoming barriers facing NPs in targeted therapy.

Many researchers reviewed the advances of microfluidic tumor-on-a-chip devices from a variety of aspects [5,28,29,30,31,32,33,34,35,36,37,38,39,40]. Tatosian and Shuler [41] devised a novel microfluidic system to study multidrug resistance of cancer cells to chemotherapeutic combinations. Wlodkowic and Cooper [42] reviewed related works regarding cancer cytomics, cell imaging techniques and micro sorting technology using microfluidic on-chip devices. Huh et al. [43] investigated the possibilities and challenges of microfluidic platforms for next generation of cell culture techniques. Zhang and Nagrath [44] investigated the works related to microfluidic in cancer study in four aspects, i.e., tumor biology, high throughput screening, cell sorting and cellular diagnostics. Young [45] reviewed varieties of design considerations of microfluidic devices used in culturing tumor microenvironment for both solid and liquid tumors. Other works [46,47] showed how researchers devised a variety of platforms to recapitulate the tumor traits such as angiogenesis, hypoxia and tumor–stromal interactions. 

The focus of this review paper is on recent investigation of tumor-on-a-chip microfluidic devices for chemosensitivity, drug toxicity and efficacy. Specifically, design, fabrication and characterization of tumor microenvironment, cell culture technique, transferring mechanism of cultured cells into the microchip, concentration gradient generators for drug delivery, in vitro screening assays of drug efficacy as well as pros and cons of each microfluidic platform used in the recent literature will be discussed separately for the tumor of following organs: (1) Lung; (2) Bone marrow; (3) Brain; (4) Breast; (5) Urinary system (kidney, bladder and prostate); (6) Intestine; and (7) Liver.

## 2. Organ-Tumor-on-a-Chip

### 2.1. Lung-Tumor-on-a-Chip

Lung is the largest material exchange with the outside environment in human body. Because outside air is breathed in, this organ is always at the risk of infection by aerosols. Lung cancer is the most common cancer and has the highest death rate among all kinds of cancers. In addition to the solid tumor, metastasis of lung malignant cells to other organs is very common [48]. For this reason, a large number of articles examined circulating tumor cells suspended in blood or culture medium either in static flasks [49,50,51] or more recently in microfluidic cell culture devices [52,53,54,55,56,57,58,59,60,61,62,63,64,65,66,67,68]. In addition, a large number of works have been carried out to identify detectors, biosensors and markers of lung cancer using a microchip [69,70,71,72,73,74]. The most common methods to culture cancer cells on microchips include: conventional 2D cell culture, 3D hydrogel encapsulation cell culture and spheroid cancer cells formation. The advantages of the last approach (i.e., spheroids) to capture tumor microenvironment has been well documented [75]. In the following parts, the works in the field of lung cancer cells on microchips based on the above methods will be reviewed.

#### 2.1.1. Conventional 2D Cell Culture

Siyan et al. [76] investigated chemotherapy resistance of the human lung squamous carcinoma cell line (SK-MES-1) by exposing it to calcium ionophore A23187 in dose-dependent manner using a concentration gradient generator (CGG) connected to eight cell culture chambers for 24 h. A23187 is a highly potent endoplasmic reticulum stress inducer which can induce the expression of glucose regulated protein-78 (GRP78) (this protein is known to be responsible for acquiring resistance of cancer cells to anticancer drugs). Then, the exposed cells were treated by an anticancer drug, VP-16, at 30 μM for 6 h. It was found that by increasing the concentration of A23187, the apoptosis rate of cancer cells treated with VP-16 decreased. These results confirmed vital role of glucose regulated proteins in chemoresistance mechanism. However, this good designed tree like CGG chip suffered from 2D cell culture weakness instead of a 3D perfect model of tumor microenvironment as well as trying cell line as an alternative to the primary human one. 

Zhao et al. [77] cultured a human non-small cell lung cancer (SPCA1) two dimensionally in a single channel microchip fabricated from polydimethylsiloxane (PDMS) and bonded permanently to a glass slide. Two groups of cells were investigated. First group was pretreated with verapamil (the calcium-influx-blocking drug) while the other group was not. 2D cultured cells were delivered with continuous medium flow to investigate the expression of P-glycoprotein under exposing to fixed concentration (30 μM) of anticancer drug, VP-16, for 6 h before being assessed via immunofluorescence. It was found that the verapamil-pretreated cells underwent apoptosis two times faster than non-pretreated cells. These results were confirmed with flow cytometry assay. Although it was the first time that cells were fed with continuous fresh medium, 2D cell culture was unable to create a real tumor environment situation as in vivo. 

**Figure 1:**
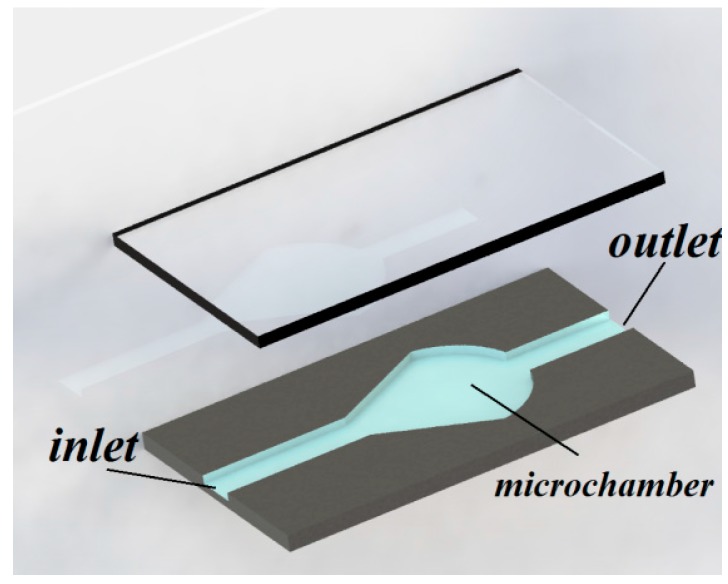
Isometric view of pear-shaped microchamber applied to decrease the fluid shear stress on cells. Reproduced after [78].

Liu et al. [78] fabricated a multilayer microdevice with a drug concentration gradient generator for cell-based high throughput drug screening to evaluate the effect of anticancer drug, cisplatin, at five different concentrations (from 0 to 91.9 μM) on cancer cell viability for 12 h. The lung cancer cell lines (A549/DDP) were cultured 2D in a static flask and loaded into microchambers via syringe pump. Then, they were trapped using pneumatically activated micropillars. In order to construct cell distribution uniformly, flow was perfused vertically and pear-shaped design was chosen to minimize the fluid shear stress on cells (Figure 1). The results successfully showed that by increasing the drug concentration, viability rate of cancer cells decreased (viability rate reduced from 90.8% to 64.2% as drug concentration increased from 9.0 to 91.9 μM).

**Figure 2:**
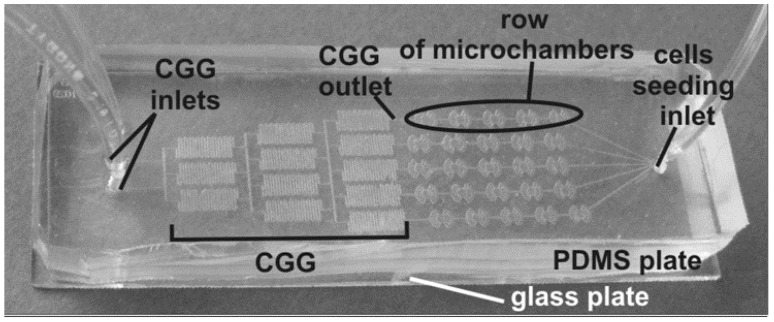
Tree-like shape concentration gradient generator provides five different drug concentrations into the microchambers [79].

Jastrzebska et al. [79] studied the behavior of the human lung carcinoma cells (A549) to experience a mixture of celecoxib (nonsteroidal anti-inflammatory drug) and 5-fluorouracil (5-FU) by a microfluidic 2D culture arrangement which included a tree-like concentration gradient generator with five different drug concentrations (Figure 2). The results confirmed the anticancer property of celecoxib by reducing cancer cell viability when combined with 5-FU. This microfluidic platform successfully showed the possibility of using multidrug combinations in antitumor activity in vitro.

#### 2.1.2. 3D Hydrogel Encapsulation Cell Culture

**Figure 3:**
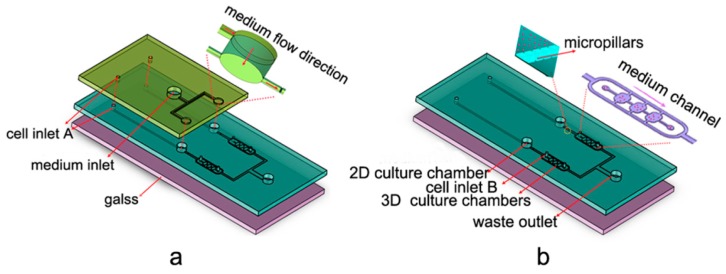
Design of the microfluidic platform involving 2D and 3D culture chambers with two layers: (**a**) top layer; and (**b**) bottom layer [80].

Hao et al. [80] co-cultured the human non-small cell lung cancer cell line (NCI-H460) with the human fetal fibroblast cell line (HFL1) in 2D and 3D Cell-gel mixture modes in a microfluidic device to investigated chemoresistance to VP-16. Using 3D spheroid enabled the desired outcomes (Figure 3). They successfully showed the mechanism of transforming the fibroblasts into myofibroblasts via secreted cytokines from cancer cells. In addition, it was found that by expressing GRP78, myofibroblasts acquired higher resistance to VP-16.

Xu et al. [81] imbedded cells in hydrogel as 3D environment in a really good designed microchip consisting concentration gradient generators system to supply different doses of drugs provided with continuous medium flow. Various types of cell lines such as the human non-small cell lung cancer (SPCA-1) and the human lung fibroblast (HFL1) as well as primary cancer cells and stroma cells from fresh lung cancer tissues were examined in mono- and co-culture conditions to investigate the effectiveness of single or mixture of anti-cancer drugs including Gefitinib, Paclitaxel and germinal center B-cell (GCB) with different dosages. Using spheroid tumor cells instead of cells imbedded in hydrogel, this can be a powerful device to be used for single patient therapy as a clinical assistance. 

**Figure 4:**
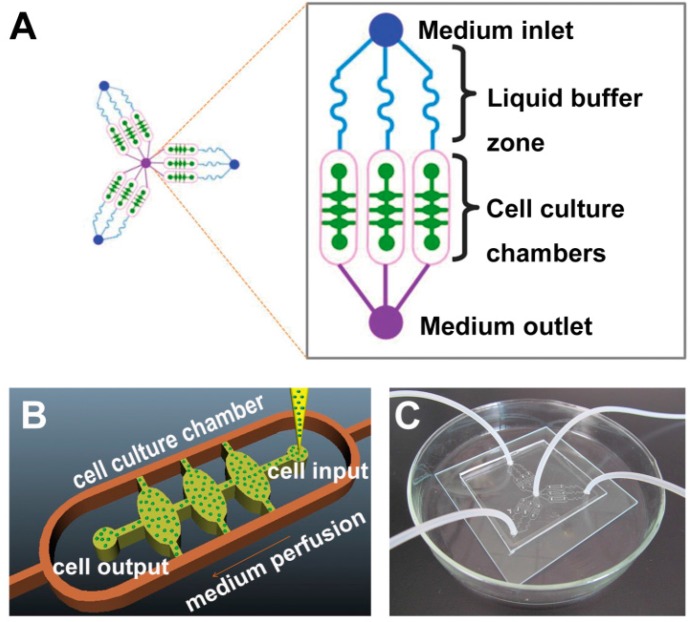
A microfluidic chip to investigate cancer cells invasion in 3D environment: (**A**) plan of the microchip; (**B**) an enlarged sketch of one of the cell culture chamber; (**C**) photograph of the device [82].

Wang et al. [82] did a deep study on invadopodia formation to investigate human lung cancer invasion using the human non-small cell lung cancer (A549) in 3D gel environmental model. To evaluate the effect of epidermal growth factor (EGF) and a matrix metalloproteinase (MMP) inhibitor, GM6001, on invadopodia formation, they fabricated a microfluidic device with one control group and two experimental groups (one with EGF and the other with GM6001/EGF), as shown in Figure 4. The results indicated that EGF promoted cell invasion while this effect was suppressed by GM6001. This signifies the possibility of using microfluidic cell culture device to discover anti-invasion drugs. 

**Figure 5:**
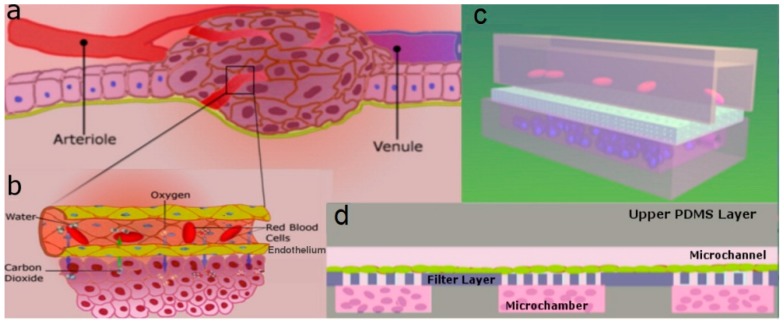
Schematic representation of: (**a**) tumor microenvironment; and (**b**) detailed view of blood vessels delivering oxygen and removing carbon dioxide. Microfluidic set up to mimic the tumor microenvironment: (**c**) 3D view; and (**d**) cross sectional view [83].

Dereli-Korkut et al. [83] fabricated a 3D microfluidic cell array that mimicked a 3D microenvironment for tumor cancer cells as well as microvascular endothelial cells to simulate blood circulation to investigate the effect of the anticancer drugs such as Tarceva (Tar), Staurosporine (Sta), TNF-α with Cycloheximide (TNF-α/CHX) and Colchicine (Col) by encapsulating the human non-small cell lung cancer cell line (PC9) and adult human dermal blood microvascular endothelial cells (HMVEC) in 3D hydrogel (Figure 5).

**Figure 6:**
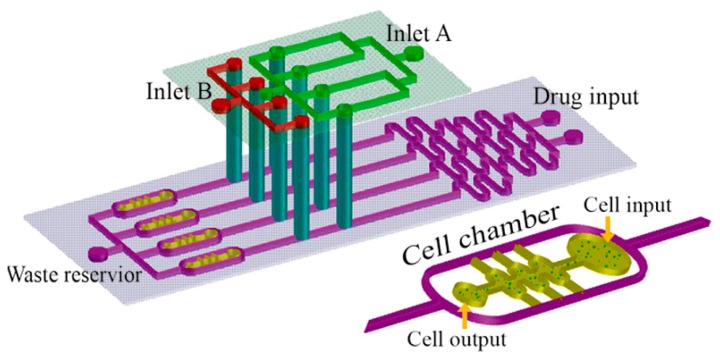
Schematic of the multi-layer microfluidic device applied to investigation the chemoresistance of cancer cells [84].

Ying et al. [84] studied the effect of hepatocyte growth factor (HGF) of cancer-associated fibroblasts (CAF) on the Met/PI3K/AKT activation, GRP78 expression on the human non-small cell lung cancer A549 cells cultured in the 3D matrix in the presence of an anticancer drug (paclitaxel). To this aim, a microfluidic device consisting of a concentration gradient generator with double-layer 3D perfusion cell culture was designed. It was used to detect the real-time cancer cells (A549) interaction with stromal cells (HFL1) and the active changes in cellular signaling along with drug responses (Figure 6). It was found that CAF matrix with HGF promoted Met/PI3K/AKT activation and triggered GRP78 expression, which hindered A549 cells apoptosis. This uncovers the mechanism underlying paclitaxel resistance and can be used to discover new combinations of targeted anti-cancer drugs. 

#### 2.1.3. Tumor-Like Spheroid Cells

**Figure 7:**
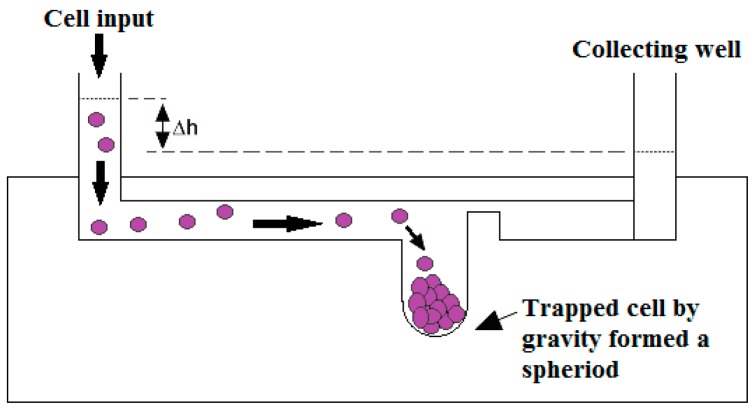
Schematic illustration of 3D microenvironment platform to generate spheroid cancer cells. Reproduced after [85].

Ruppen et al. [85] fabricated round bottom microwells using PDMS as 3D microenvironment to construct spheroid of cancer cells, as shown in Figure 7. Suspended cells were transferred into the microchips through the inlet channel using hydrostatic pressure and trapped in the microwells due to gravity. Cell viability and proliferation test was done successfully. Both mesothelioma cell line (H2052) and primary patients (non-small cell lung adenocarcinoma and pericytes) mono- and co-culture were used and the effect of stromal cell on the drug resistance was investigated. Anticancer drug resistance was evaluated using perfused cisplatin through medium channel. This was an important contribution in cell spheroid generation with medium perfusion for chemosensitivity assay. However, adding a concentration gradient generator system to deliver various dosages of drugs can make it more realistic.

### 2.2. Bone Marrow-Tumor-on-a-Chip

**Figure 8:**
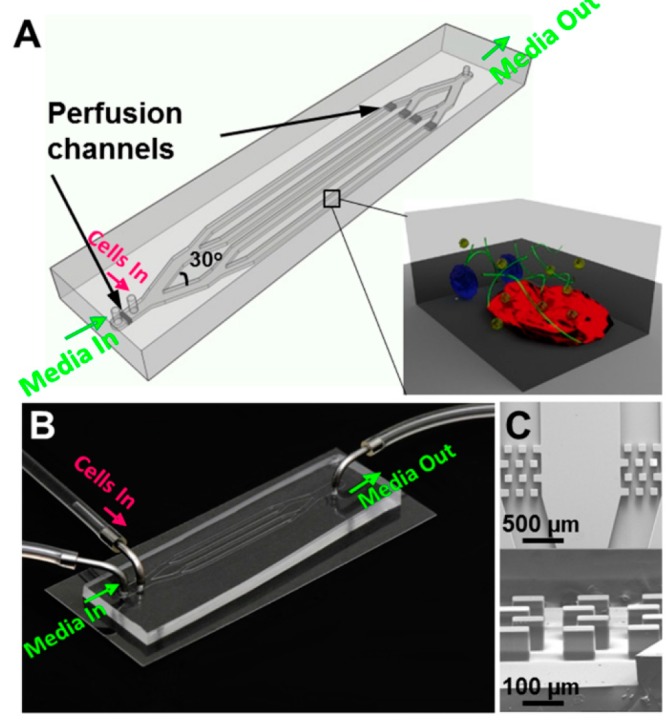
Schematic (**A**,**B**) and scanning electron microscope (SEM) images (**C**) of the microfluidic platform used to study acute lymphoblastic leukemia [86].

Bruce et al. [86] studied acute lymphoblastic leukemia (ALL) in 2D and 3D static microenvironments as well as 3D dynamic microenvironment. As this type of cancer initiates in the bone marrow and then develops, they cultured a mixture of these cancer cells with primary bone marrow stromal cells and osteoblasts. Following the standard photolithography followed by PDMS soft lithography techniques, the microchannels were fabricated and bonded on glass cover slip. Several rows of columns were created near the inlet and outlet to retain the cells within the main microchannels. Seventy percent ethanol and UV exposure were used to sterilize the microdevice. After loading the cells with culture medium into the microchannels, collagen was gelatinized using incubation and then in 3D dynamic model, the culture medium was pumped continuously to microchannels using a syringe pump. They studied chemoresistance to antimetabolite chemotherapeutic drug (Ara-C), which is widely used as a therapeutic agent in the treatment of blood cancers, for different conditions such as 2D and 3D, mono- and co-cultured and dynamic and static models. For this purpose, Ara-C was diluted with culture medium and after incubation; the cells were exposed to the drug for 48 h. They used flow cytometry and immunofluorescence image analysis to examine cell viability. Their results showed that the survival of leukemic cells in the tri-cultured system during chemotherapy for all three models (2D and 3D static and 3D dynamic microenvironments) was more than the monoculture system. The microenvironment protection in 3D dynamic model was more than 3D static model and the 3D static microenvironment protection was more than 2D static model. They attributed the difference in chemoresistance between 2D and 3D models to difference in cell-matrix interaction, which was controlled by integrin activation and cell–cell signal transduction. They introduced collagen matrix as an obstacle that limited drug delivery. This system is shown in Figure 8.

Young et al. [87] investigated the effect of chemotherapeutic drugs (bortezomib and TNF-α) on multiple myeloma (MM) cells. Soft lithography was used to create the microdevice consisting of a central chamber and side chambers, which were connected by radial diffusion ports. They sterilized the system by 70% ethanol. RPMI 8226 cells (human MM cell line) and HS-5 cells (human bone marrow stromal cell line) were cultured and dispensed in center chamber and side chambers, respectively. One of the positive features of their microfluidic system was that it retained cells in microchannels without any cell traps. This feature reduced the limitation of the spatial distribution of the cells and allowed them to freely proliferate or migrate. Less pressure drop in this system compared to the systems with cell traps enabled them to use passive pumping for volume replacement to feed, deliver and provide immunostaining. They showed that by increasing cell culturing time before volume replacement, cell retention can be improved. With 16 h incubation more than 99% of the cells were retained. Bortezomib as a proteasome inhibitor for treatment of multiple myeloma (MM) cells was directly injected to microchamber and then fluorescence based cell viability assay was used to investigate the chemoresistance of the stained cells. They observed that by increasing drug concentration, cell viability was decreased in a dose-dependent manner. They also used microfluidic single cell nuclear translocation (μSCeNT) assay to study activation of NF-κB and STAT3 as transcription factors which have a critical role in the regulation of important cellular process in cancers. They dose-dependently examined NF-κB activation and inhibition after cytokine and drug delivery and also observed activation of STAT3 in MM cells in the co-culture model with bone marrow stromal cells (BMSCs). 

Pak et al. [88] investigated chemoresistance of MM cells cultured cis-co-culture in the microfluidic device which were used in their previous work [87]. To study the roles of cancer and non-cancer cells in response to chemotherapeutic drug (bortezomib), they dispensed MM cells in the central chamber and patients’ own non-MM cells (such as BSMCs and macrophages) in the side chambers. This method of culturing cell in separate compartments allowed interaction between MM and non-MM cell suspensions, but did not include the effects of direct contact of these types of cells. After the cells were treated with bortezomib, to identify dead and live cells, they were stained. With this method, they were able to divide the patients into two groups of responsive and non-responsive to the drug that was consistent with the clinical results, while monoculture model found to be less accurate.

### 2.3. Brain-Tumor-on-a-Chip

Glioblastoma Multiforme (GBM) is the most malignant form of brain tumor, and has a median survival time of 12–15 months. Different microchips have been developed to culture brain tumor. 2D cell culture is more common for its easiness but has limitations to reconstruct behaviors, which are physiologically similar to live tissues. For this reason, cell spheroids and tissue culture have recently emerged to better recapitulate the microenvironment of cells in vivo. However, delivering the nutrients and gas exchange are the challenges for 3D cell culture devices.

**Figure 9:**
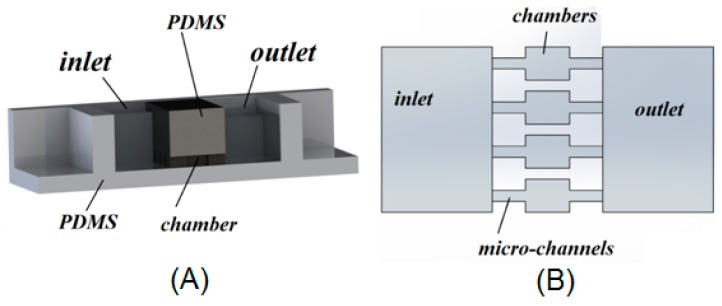
Isometric (**A**) and top views (**B**) of microfluidic system designed for rat glioma cell culturing. Reproduced after [89].

One important application of microfluidic system for brain tumor is the development of anticancer agents for brain tumor therapy. Liu et al. [89] used a microfluidic device with four parallel chambers fabricated using PDMS soft lithography to culture rat C6 Glioma cells, as shown in Figure 9. The cells were cultured with a constant flow of Dulbecco’s modified Eagle’s medium (DMEM) and Fetal bovine serum (FBS) at a low hydrostatic pressure. Cellular response to different concentrations of colchicines (0.05–10 μg/mL) was tested after 48 h culturing. In order to maintain acceptable cell growth and proliferation, cell response study was performed in a short-term cell culture manner (2 days). Cell viability was visualized by fluorescent images after Propidium iodide (PI) staining. They observed significant morphological changes and cell death rate by increasing Colchincine concentration or treatment time.

In another research, Ayuso et al. [90] treated U-251-MG cells within collagen hydrogel with different drugs such as anticancer drug cytotoxic T lymphocytes (CTL), anti-proliferative agent temozolomide (TMZ) or hypoxia activated prodrug tirapazamine (TPZ). To enable visualization of microenvironment, viable/dead cells were labeled with Calcein/PI. This microdevice could be a useful platform to test chemosensitivity of tumor cells against new anticancer agents in preclinical phase.

**Figure 10:**
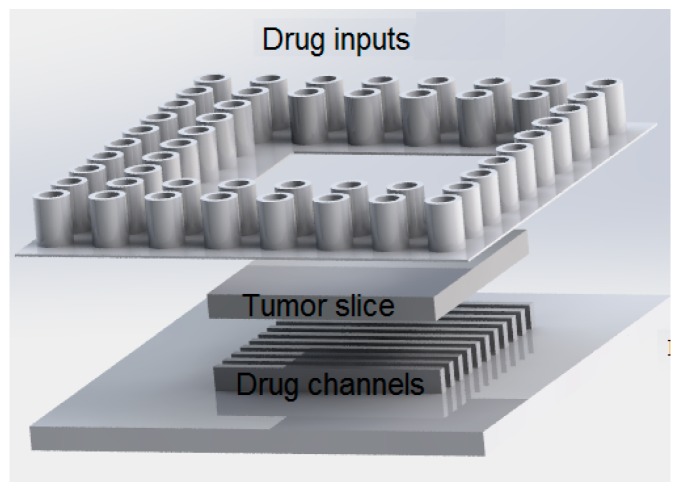
Schematic representation of the system used to assay the response of tumor slice to an anticancer drug (temozolomide). Reproduced after [91].

Chang et al. [91] used human Glioblastoma cell line GBM8 to evaluate the response to TMZ. The most prominent distinct of this work with others is the use of tumor tissue instead of tumor cells. Glioma cells were injected into a mice brain and allowed to grow. Then GBM xenograft slices with the thickness of 400 μm were prepared. They fabricated a microfluidic device using multilayer PDMS soft lithography. Tissue slices were cultured on a Poly-tetrafluoroethylene (PTFE) porous membrane. The membrane from bottom was connected to a reservoir well of 96-well plate by different microchannels as shown schematically in Figure 10. In order to understand drug delivery in tissue slice, a simple diffusion-based model using COMSOL Multiphysics^TM^ was employed and it was experimentally visualized by fluorescent cell tracers. In their microfluidic platform, the drug channels were separated by buffer channels to prepare variable doses of drug to deliver it to tissue slice. Dose-dependent chemosensitivity profiles were exploited using staurosporine (STS) fluorescence cells. The results showed the feasibility of quantifying readouts of fluorescence images from different regions of tissue with different doses of drug exposure. This research can be useful in screening chemotherapeutic drug for fast decision-making at initial phases of cancer therapy. 

It has been proposed that deficiency of nutrients and oxygen causes the glioma aggressiveness. Consequently, by migration of tumor cells towards nutrients and oxygen enriched region, GMB pseudopalisades will be formed. Ayuso et al. [92] studied the process of generating pseudopalisades in Glioblastoma using microfluidics. They cultured GBM cells (U-251-MG) within a SU-8 based microdevice [93] with controlled flow through lateral microchannels and mimicked nutrient starvation circumstances. Their results showed that in early stage when nutrients were sufficient, GBM cells had non-invasive behavior. However, after depletion of nutrients, GBM cells initiated to migrate toward enriched cells, causing pseudopalisades to be generated. 

Standard diagnostic methods of solid tumors are immunohistochemistry (IHC). Unfortunately, quantification of IHC data is restricted by different variables and data analysis is challenging. This, is not the case for flow cytometry, but sample/reagent consumption is high. Thus, researchers have focused on a miniaturized platform to make molecular diagnosis of solid tumors more economic and sensitive. For example, Sun et al. [94] reported a microfluidic image cytometry (MIC) for single-cell proteomic analysis using cultured U87 (human glioblastoma cell line). They measured four critical signaling proteins (EGFR, PTEN, phosphor-Akt and phosphor-S6) simultaneously by a PDMS microchip comprised of 24 cell culture chambers. In order to establish on-chip immunocytochemistry, immunolabeling was performed by fluorophore-conjugated antibodies and image acquisition was processed by MetaMorph software. The developed MIC measurements were validated by IHC clinical tests. Bioinformatic methods, such as self-organizing maps, were also adapted to compare multiparameter MIC data sets with clinical measures.

The migratory behavior of brain tumor stem cells was studied by Haung et al. [95] who used a PDMS microchip consisting of three connected compartments: seeding chamber, receiving chamber and bridging microchannels. They reported that the migrating cells experience sequence of six stages based on their morphological changes. Important aspects of their work were live imaging in one box for several days, which avoided any disturbances and using serum-free stem cell medium as neurospheres.

Recently, microfluidic electrophoresis system has played an effective role in authentication of brain tumor cell lines. Qian et al. [96] investigated reliability of DNA profiling of different cell lines using microfluidic-based electrophoresis platform. They also evaluated the reproducibility and consistency of DNA profiling over multiple subcultures. This method was proven to be simpler and more effective for FLA (fragment length analysis) than capillary electrophoresis. It was also efficient for sizing and quantifying DNA, RNA, proteins, and cells.

### 2.4. Breast-Tumor-on-a-Chip

To analyze cytotoxicity of human breast cancer cells, Kim et al. [97] developed a microfluidic device with microwells from PDMS, which were attached together using air plasma treatment. Hydraulic resistance of microchannels was regulated to obtain equal flow rate in each microchannel. They cultured MCF-7 (human breast cancer cell) cells to examine cytotoxicity in treatment by mitomycin C (MMC), 5-fluorouracil (5-FU) and doxorubicin (DXR). Cancerous cells were injected into microdevice by a syringe pump. Flow rates were regulated as specific numbers of cells were trapped in the wells to form uniform spheroids. The microwell wall was coated with bovine serum albumin (BSA) to avoid cell attachment. The cells deposited in trapped wells, but did not attach to the wall. They aggregated and formed tumor spheroids. They cultured the cells in hanging drop (HD), in their 2D model and compared the results. They observed that the ratio of tumor spheroids in microwells was more than HD and the spheroid diameters in microwells were uniform. In 3-(4,5-dimethylthiazol-2-yl)-2,5-diphenyltetrazolium bromide (MTT) assay by spectrometer, optical density was measured. This method required large samples, so they presented a new test based on color intensity measurement and compared the results with the results of MTT assay. The results were in good agreement with each other. They investigated cytotoxicity of MMC, 5-FU and DXR in both 2D (monolayer culture) and 3D (microfluidic device) models and observed the inhibition rate with increase of drug concentration and treatment duration decreased. In the 3D model, the inhabitation rate was much less than the 2D model, because in the 3D model cell–cell interaction in aggregates was extensive. They reported that MMC in the treatment of this cancer was more successful. Hwang et al. [98] used a simple yet effective technique (multilayered adhesive tapes) to fabricate a master mold for PDMS soft lithography of microfluidic cell culture platform. Both monoculture of a breast cancer cell line (MCF-7) and co-culture of MDA-MB-231 with NIH/3T3 fibroblasts were successfully done in the device and chemosensitivity assay of an anticancer drug (Tamoxifen) was conducted accordingly. Breast cancer metastasis to other organs (bone) was also evaluated by fabricating a 3D microfluidic system [99]. 

### 2.5. Urinary System-Tumor-on-a-Chip 

The urinary system of the human including kidneys, ureters, bladder, prostate and urethra is one of the important systems of body which eliminates wastes, regulates blood volume, and performs lots of other vital functions. Different types of cancers observe in different organs of urinary system but since the prostate and bladder urothelial cancers are more prevalent, more groups have focused in the prevention and therapy of these two cancers.

Lin et al. [100] used microfluidic chip to quantify a urine bladder cancer biomarker, APOA1. In that work, a suction-type (negative pressure) micro-mixer and micro-valve were used. In addition in order to supply the primary antibody, magnetic beads were used. A four-layer, three-dimensional (3D) microchip was fabricated. It consisted of two air layers for controlling mixer and valves and an internal layer for fluidic part and a PDMS layer for sealing the microchip. The measurement results were compared with classical 96-well conventional enzyme-linked immunosorbent assay (ELISA) and good agreement was obtained. The most important benefit of the microchip was the faster detection of the cancer cells compared to previous systems. In addition, fabricating an exact three-dimensional microchip with thorough details was the proscenium aspects of the work; yet, tumor microenvironment evaluation of the beads was not discussed. 

**Figure 11:**
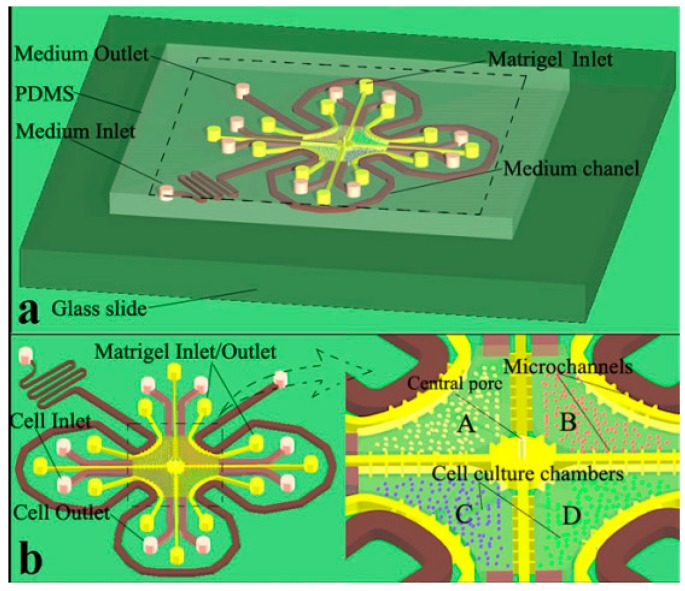
(**a**) Geometry of the chip in bladder cancer therapy; (**b**) Magnified view of cell culture chambers where bladder cancer cells are cultured in indirectly connected central chambers (A–D) [101].

Liu et al. [101] fabricated a microfluidic system to simulate the bladder cancer microenvironment. In order to construct the three dimensional microenvironment, bladder cancer cells (T24), with the stromal cells, fibroblast, endothelial cells, and macrophage were used. Perfusion equipment, matrigel and different channels used in order to optimize accessibility to the four types of cells. The microfluidics layout is shown in Figure 11.

In this experiment, the influence of different clinical neo-adjuvant chemotherapies on bladder tumor was studied. According to the paper’s results, MVAC group (methotrexate, vincristine, doxorubicin and cis-Diammineplatinum Dichloride) is more sensitive chemotherapeutic candidate to treat bladder cancer cells. Although tumor microenvironment was captured satisfactory in this work, the complexity of their design and difficulties in system control were disadvantages of the device toward commercialization.

The work done by Lie et al. [101] competed in 2015 by Zhao et al. [102]. They used the same microfluidics and a similar microenvironment to study the influence of lactate shuttling on macrophage and tumor cell behavior in the bladder tumor microenvironment. Although the difficulties in fabrication process also existed in that work, it involved a diversity of drug tests, which could be mentioned as the strength of the work.

Prostate cancer is also another urinary system’s widespread cancers whose early diagnosis can be very helpful in treatment. Using lab-on-a-chip technology, vast amounts of works have been done in detection, separation, and therapy of the prostate tumor. Salmanzadeh et al. [103] discovered a dielectrophoretic response of prostate initiating cells to electric field. Tumor initiating cells were cultured spherically—in a three dimensional microenvironment—and allowed to pass through a pillar based microchip. They demonstrated that the prostate cells (PC3) made larger spheroids in the dishes with no electric field compared to those released in 280 Vrms. In addition, they were able to show that the spheroid started to get larger in the presence of electric field. The idea of using electric field to make tumor smaller can be very useful in cancer therapy, but the work could have been more valuable work if it had compared the obtained results with the drug tests. In addition, tracing the cancer cells might be needed after decomposing the tumor which might increase the possibility of metastasis.

Comparing the previous works, it could be concluded that some limited works have been done in bladder and prostate tumors using microchips but the shortage makes sense in kidney and other organs of urinary system. In addition, most of the works have been done in cancer detection. As a result, large amount of works are still needed in drug screening as well as personalized medicine. 

### 2.6. Intestine-Tumor-on-a-Chip

Chen et al. [104] presented a new microfluidic system containing the rows of cylindrical microwells in microchannels. These microwells allowed parallel loading of different cell types and used different drug concentrations. For microdevice fabrication, they used soft lithography and rapid prototyping and for sterilization, they used 70% ethanol and UV exposure. After injection of cancerous cell into microchannels, the cells in microwells aggregated and finally created tumor spheroids. They used poly(vinyl alcohol) (PVA) to avoid attachment of the cells to the walls. The growth of spheroids was observed by optical microscope. The results showed that until the sixth day, the cells proliferated and the spheroid diameter became larger and then remained almost constant. They chose colon cancer cells (HCT116), breast cancer cells (T47D) and hepatocellular cancer cells (HepG2) to analyze the effect of therapeutic agents, doxorubicin (DOX) and paclitaxel (PTX), on tumor spheroids by in situ live/dead fluorescence staining assay and MTT assay. Their results showed that by increasing drug dosage, cell viability decreased. According to their results, in a high dosage of DOX, the dead cells were placed in the center of spheroids for all three tumor types and HepG2 spheroids lost their structural integrity; however, in low dosage, the dead cells were located in the surface of spheroids. The effect of PTX on cell viability of HCT116 and HepG2 cells was less than DOX and just on the surface of these spheroids, the dead cells were observed, but it destroyed T47D spheroids. They repeated their experiments for different sizes of HCT116 spheroids and reported that by increasing the size of tumor spheroids, sensitivity to DOX and PTX was reduced. Therefore, anticancer drug efficiency is closely dependent on the conditions of cell growth. They also indicated that tumor spheroids were more resistant to drug treatment than the 2D cultured models.

### 2.7. Liver-Tumor-on-a-Chip

Liver, as massive and main metabolizing organ in the body, has an intricate structure comprised of two primary cell groups; videlicet parenchyma cells called hepatocytes (functional cells constitute 80 percent of all liver cells) and non-parenchyma ones including endothelial cells of sinusoid lumens, stellate cells (active in blood microcirculatory of sinusoids), Kupffer macrophage cells (moving on the inner surface of sinusoids to clean up intestinal bacteria and dead blood cells), pit cells (with same location of Kupffers to disrupt and perish circulating tumor cells (CTC)). According to essential roles of hepatocyte cells such as protein synthesis/storage, metabolism of carbohydrates, cholesterol, phospholipids and bile salts, bioengineers have focused on them to investigate biological and functional behaviors of liver in in vitro systems. Survival of normal hepatocytes (mature primary hepatocytes or pluripotent stem cells) depends on high oxygen concentration and specific shear stress. Therefore, hepatocarcinoma or hepatocyte cancer cell lines (HepG2 or its clonal derivative that has strong contact inhibition of growth named C3A) require more strict environmental conditions to survive longer time in vitro. 

In drug development and screening, cytotoxicity assay is vital. It can predict toxic organ (either liver (hepatotoxicity) or other organs (hematological toxicity)) due to drug consumption itself (direct toxicity) or hepatic-generated metabolites of drug (side-effect toxicity). Adverse effects caused by a drug metabolite rather than the drug itself, increase hepatocyte contribution in cell-, tissue-, and organ-on-a-chip concepts. Two major examples of liver contribution to drug metabolism are inversion of prodrugs such as thymosin fraction 5 (TF5) to metabolite drugs like fluorouracil (5-FU) that causes hepatotoxicity and metabolism of doxorubicin to doxorubicinol that causes hematological toxicity (such as cardiotoxicity or bone marrow toxicity). Accordingly, to investigate the pharmaceutical aspects of anticancer drugs, liver needs to be co-cultured in microscale cell culture analog (µCCA) systems [105,106]. For instance, following works co-cultured liver in their µCCA systems: liver–uterine–bone marrow [41], liver–heart–vascular [107], liver–intestine–breast [108], liver–prostate–kidney [109], liver–breast–cervical [110], liver–lung–fat [111], liver–lung–kidney–fat [112]. These co-culture systems have mostly utilized homotypic hepatocarcinoma cells (including pure hepatocyte cell–cell interaction in liver chamber) to investigate mutual effects of liver and other organs cultured in separate chambers linked together by microfluidic channels. According to metabolize cascade between liver and intestinal system [113,114], their co-culturing called liver–gastrointestinal system is strictly suggested particularly in studies of oral-drug development [115]. These systems mainly aim to consider absorption, distribution, metabolize, and excretion (ADME) processes. Most liver co-culture µCCA systems fabricated to evaluate the efficacy of anticancer drugs of non-hepatic cancerous targets, such as lung, colon, kidney, and bone marrow cancers, employ resistant hepatocarcinoma cells instead of healthy primary liver cells. This underestimates the drug side effect on the liver since liver cancer cells have very high (low) viability (apoptosis) rate. The requirement of culturing primary hepatocyte cells in µCCA systems is more pronounced in evaluating non-hepatic multidrug resistance (MDR) of cancers with drug/modulator mixtures. Since MDR tumor (as target) firmly needs to be distinguished from healthy co-cultivated tissues when evaluating the efficacy of combinations of drugs [41]. Sensitivity of liver metabolizing to dose of drugs offers the development of drug gradient generator platforms created with certain architecture of microfluidics to regulate drug doses in the co-culture systems. In addition to drug dose, physical properties such as pressure and flow rate, substances concentration, and loading ratios (cells to organ/flow to cells ratios) contribute in the liver activity and metabolism [116]. 

**Figure 12:**
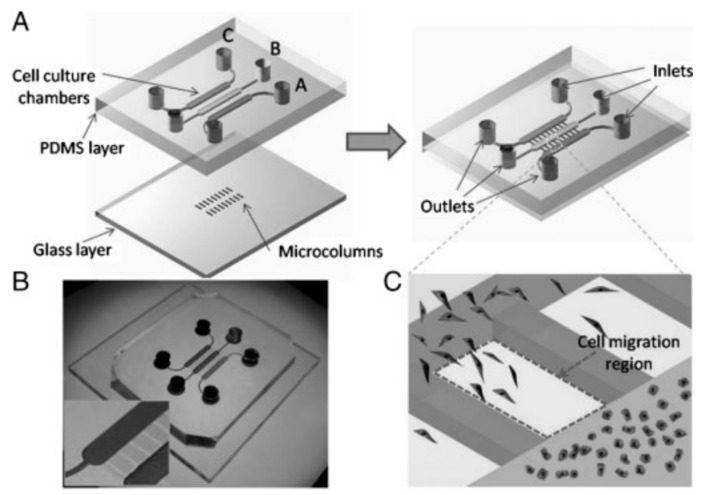
Interaction of hepatocarcinoma and fibroblast cells in a microfluidic device: (**A**) layout of cell co-culture chambers with parallel regions to capture migrating cells; (**B**) photograph of the microfluidic device; (**C**) magnified image of the cell migration through parallel regions [120].

Because of more sensitivity of hepatocarcinoma to environmental conditions compared to other cancer cells, tumor microenvironment must be accurately recapitulated. High cell–cell interaction in the in vivo microenvironment leads to exploiting of 3D hepatocarcinoma cell culture in many recent works instead of conventional 2D monolayer cultures. Major challenge facing 3D hepatocyte culture is how to promote hypoxia and necrosis of surrounded internal cells in a functional realistic three-dimensional structure. Recently, 3D spherical cultures with hanging drop network (HDN) of medium to incubate hanging spheroid tissues (without cell-surface adhesion) [117] advance towards more functional and biological microenvironments of cancer than 3D conventional ones with cell-surface adhesion. It is more convenient to evaluate drug cancer resistance, cancer metastasis, and angiogenesis process. High gas exchange (O_2_, CO_2_) during incubation, prohibition of bubble formation during medium secretion and cell injection can be considered as HDN advantages in comparison to usual 3D cultures. In addition oxygen-permeable chips promote hypoxia and necrosis of central hepatocarcinoma cells in 3D spheroid models [118]. Naturally, the cancer angiogenesis itself helps to receive oxygen and nutrients to central surrounded cells. It has been proven that 3D heterotypic spheroid models with more realistic configuration have drug resistance rather than 2D homotypic ones. It increases cancer cell viability and proliferation towards generation of huge spheroid tissue [119]. However, in the absence of anticancer drugs, 3D spheroids have proliferation rate less than 2D monolayers. Heterotypic hepatocarcinoma models with stromal compartments namely extracellular matrix (ECM) substances, stromal fibroblasts, and immune cells can be considered as cancer–stromal interaction models to fabricate proper cancer microenvironment [119,120]. Other heterotypic models include hepatocyte and endothelial cells to recapitulate real structure of sinusoids to provide accurate hepatocyte behavior [121]. However, heterotypic models including other probable functional liver cells such as Kupffer macrophage and pit cells can be investigated in future works on circulating tumor cells (CTC) and metastasis cascade (invasion, intravasation, extravasation, secondary tumor). Metastasis cascade investigation to predict secondary tumors in the µCCA systems could be very challenging subject to anticancer drug development as well as MDR treatments. Briefly, using µCCA model (as co-culture model) with 3D hetero-spheroid culture (Hep G2/C3A with the other liver cells and stromal compartments) can be best candidate to investigate drug resistance and metastasis of solid cancers. Figure 12 illustrates a microfluidic co-culture device to capture interaction of hepatocarcinoma and fibroblast cells [120]. This is a simple 2D case to recapitulate the tumor microenvironment that can be used in a comprehensive µCCA system to interact with other organs. 

## 3. Multi-Organ Co-Culture in Tumor-on-a-Chip 

In previous section, we emphasized on the importance of liver co-culture μCCA systems for anticancer drug screening applications. Co-culturing other organs such as intestine, lung and kidney parallel to the target cancerous organ can better recapitulate human physiology for in vitro drug screening and development. In drug discovery, physiologically based pharmacokinetic (PBPK) and pharmacodynamic models offered by multi-organ co-culture μCCA systems can be very useful. Further, this platform is especially important for pharmaceutical industry in clinical trials. After drug discovery and development, there are many costly and time consuming phases before it can enter into the market to be used by patients. Success rate of most newly discovered drug in clinical trials is extremely low (10%–15%) [122]. This is a huge loss for pharmaceutical companies since total phases of clinical trial cost over US$15,000 per patient. Therefore, multi-organ co-culture μCCA systems, which are also called “body-on-a-chip” or “human-on-a-chip”, can be vital prescreening phase to more accurately predict drug efficacy and its side effects on other organs. Interested readers are encouraged to refer to other invaluable works, especially those conducted by Ingber and colleagues [123] at Wyss Institute at Harvard University as well as Shuler et al. [124,125,126,127] at Cornel University. In the following part, the importance of heart–liver co-culture, which we call “cardiohepatic”, as well as blood vessels in tumor-on-a-chip application will be further discussed. 

### 3.1. Cardiohepatic Interaction in Tumor-on-a-Chip

Cancerous tumor of heart (sarcoma type of cancer) is so scarce that can be ignored in tumor diagnosis and therapy researches as well as tumor-on-chip assays. Since there are vital interaction between contractible cardiomyocyte cells and hepatocyte cells, heart must be an inseparable component from the µCCM systems involving hepatocarcinoma or hepatocyte cells (the necessity of heart–liver co-culture). Other heart cells, such as cardiac-fibroblasts and vascular smooth muscle cells, can also be utilized to fabricate heterotypic heart culture. On the one hand, clinical observation suggests that heart failure (HF) can lead to lack of vital metabolites for other living organs including liver. On the other hand, metabolism activity of liver can cause the side-effect toxicity of cardiomyocytes (cardiotoxicity) [128,129]. This metabolism circulating in toxicology field suggests that cardiohepatic synergetic (CHS) system is vital to study cytotoxicity of anti-hepatocarcinoma drugs (such as 5-FU and DOX), mutual effects of liver-generated drug metabolites and HF dysfunctions. Therefore, anticancer drug failure due to inaccurate profiles of absorption, distribution, metabolism, and excretion (ADME) as well as toxicity can be addressed using CHS systems that approximately complete ADME puzzle with metabolism and distribution pieces. Two remaining pieces of puzzle, absorption and excretion, can be inserted by coupled gastrointestinal system to CHS. Note that gastrointestinal in parallel to liver also plays a key role in the metabolism cascade via intestinal enzymes. Summarily, µCCM systems involving heart–liver–intestine co-culture (cardio-hepatic-synergetic-gastrointestinal (CHSGI) systems) would be the best candidate to authentically develop anticancer drugs and clarify human responses to drugs. In addition, HF and cardiotoxicity may also occur by anticancer drug itself when co-cultured with other cancerous organs, such as lung, kidney, and breast. Therefore, heart must be considered in complex µCCM systems to obtain authentic results of direct and side-effect toxicities.

### 3.2. Microvasculature

To realistically fabricate tumor microenvironment, formation of endothelial-lined blood microvascular system, which allows tumor angiogenesis in carcinoma tissues, is a major factor to consider [45,107,130,131]. Indeed, the microfluidic channels linking micro-tissues in a µCCM can be covered by blood vessel endothelial layers more accurately resemble in vitro circulatory system. It has been illustrated that tumor angiogenesis occurs primarily in physiological microchips consisting endothelial-lined microfluidic channels instead of conventional ones. If microfabricated chip provides suitable environment for angiogenesis activity, tumor spontaneously supplies its requirement of oxygen and nutrient particularly for central cells trapped in huge 3D spheroids and accordingly addresses hypoxia and necrosis challenges. Therefore, appropriate tumor microenvironment leads to form huge and resistant 3D cancer structures, which are more suitable for anticancer drug screening. Therefore, microvascular network can be the other inseparable component of multi-organ co-culture μCCA systems for chemosensitivity assay.

## 4. Conclusions

Cancer is a genetic disease caused by mutations in genes of the cells. Since individual’s genome is unique, tumor response to anti-cancer drugs may differ from one patient to another. This concept, which is closely linked with the emerging field of “personalized medicine”, can be best addressed using microfluidic platform. It can replace traditional 2D/3D cell culture in vitro techniques for drug screening and development. This platform, also called microscale cell culture analog (μCCA), can be considered the next generation of pharmacokinetics and/or pharmacodynamics models. In cancer research study, it can be a very helpful tool with potential of providing patient-specific therapy. It can provide a more realistic environment for chemosensitivity assay, evaluating the effectiveness of nanoparticles (NPs) in targeted drug delivery, understanding the possibility and potential of multi drug resistance (MDR) as well as cancer metastasis. 

In this review article, we focused on the first application of μCCA, by reviewing the works related to the fabrication of microfluidic platforms to evaluate the efficacy and toxicity of anticancer drugs. In particular, it was categorized based on the tumor organ. The following important features of organ-tumor-on-a-chip devices were discussed in detail: (1) selected materials; (2) tumor microenvironment; (3) cell culture technique; (4) transferring mechanism of cultured cells into the microchip; (5) concentration gradient generators (CGG) for drug delivery; and (6) in vitro screening assays of drug efficacy. The results are summarized in Table 1. The importance of co-culturing multi-organ in tumor-on-a-chip devices was also emphasized. Specifically, it was shown that heart–liver–intestine co-culture tumor-on-a-chip devices with blood vessel endothelial layers (microvasculature) can be the best candidate for in vitro chemosensitivity assay. Such systems would complete ADMET of anticancer drugs and more realistically recapitulate the tumor in vivo-like microenvironment.

## Figures and Tables

**Table 1:** Organ-tumor-on-a-chip.

Organ	Reference	Selected Materials	Tumor Micro Environment	Cell Culture Technique	Transferring Mechanism	CGG	In vitro Screening Assays
Lung	[85]	PDMS	Mono- and co-culture of mesothelioma cell line (H2052) and primary patients HNSC	Tumor like Spheroid formation	NMC	-	CellTrace™ CFSE Cell Proliferation Kit
	[83]	PDMS	HNSCLC cell line (PC9) ccwHDMEC)	EH	NMC	-	Calcein AM
	[78]	PDMS	LC cell line (A549/DDP)	2D cell culture	NMC	Five steps of linear CCG	Fluorescence probe DAPI
	[79]	PDMS/Glass	Cell line A549	2D cell culture	NMC	Tree like steady state	MTT
	[80]	PDMS	Co-cultured HNSCLC (NCI-H460) and HFFCL(HFL1)	2D and 3D Cell-gel mixture	NMC	-	Trypan blue exclusion assay
	[81]	PDMS	Various types of cell lines	EH	NMC	-	MTT
	[82]	PDMS/Glass	HNSCLC (A549)	EH	NMC	Simple network	IFSA
	[84]	PDMS	Human LC A549 and fibroblast HFL1 cells	EH	NMC	Tree like steady state	IFSA
Brain	[89]	PDMS	Rat glioma cell interaction	Using hydrostatic pressure	-	Injection of specific drug	PI staining
	[90]	Polystyrene-based	TSI	Suspending in hydrogel	-	Injection of specific drug	Calcein/propidium iodide
	[91]	PDMS + PTFE	Tumor Slice			Exposing to drug in a specific location of tissue	Caspase-3/7 and eFluor 660
	[92]	SU-8	Cells embedded within a hydrogel	Suspending in hydrogel	-	No concentration gradient	FDA and PI
	[95]	PDMS	Cells embedded within a hydrogel	Suspending in hydrogel	-	-	-
Urinary System	[100]	PDMS/Glass	TSI	EM	NMC	-	IFSA
	[101]	PMMA/PDMS	-	-	-	-	Enzyme-linked Immunosorbent assay (ELISA)
	[102]	PDMS/Glass	TSI	EM	NMC	Diffusion base	-
							Cell immunoflorescence
							Western blotting
	[103]	PDMS	-	Suspended spheroid colonies	Cells are located inside the main fluid	-	Prostasphere assay
							Aldefluor assay
Liver	[95]	HDM platform	3D Tumor spheroid/homotypic culture	HDT for 96-well plate	Multi-channel pipette	-	LDH and ATP
	[132]	PDMS and collagen	3D Tumor/homotypic culture	3D mFCCS	Withdrawal syringe pump	-	ATP (*Calcein AM based*)
	[120]	PDMS	2D TSI	2D culture/ccw fibroblasts	Cell suspension injection	-	ATP and SMA
	[127]	PDMS	3D Tumor/homotypic culture	EM/co-culture	Direct cell culture in the specific chambers	-	MTS
	[119]	Polystyrene/Collagen hydrogel	3D heterospheroid and 2D homospheroid TSI	HDT and EH	Micropipetting cancer/stromal cells	-	EROD and Alamar blue
	[118]	Polystyrene/PDMS	3D tumor spheroid	6-well plate	Plastic pipette	-	DNA
	[108]	PDMS	2D tumor/homotypic	Glass and bead-base culture	Injection of cell suspension	Manually syringe pump	Redox-based assay (ATP)
	[41]	Silicon (PDMS)	2D tumor/homotypic	2D cell culture	Injection of cell suspension)	Steady drug compotation	MTS
							DNA
	[110]	PDMS	Tumor Invasion and Metastasis	2D 6-well plate	Gravitational flow	-	TBE and CMI
	[117]	PDMS	3D tumor spheroids/homotypic	HDT for 16-well plate	Single pipetting	Tree-like steady state	ATP
	[133]	PDMS	2D tumor/homotypic	2D 6-well plate	Injection of cell cultured	-	CYP1A activity
	[134]	PDMS	2D tumor/homotypic	Parallel cell culture chambers	NMC, steady state	Multiple drug gradient generators	DNR, IDA, DDP, CBP, MMC, BLM, and ActD
	[112]	PDMS	3D tumor/homotypic	3D μFCCS/co-culture	Withdrawal syringe pump	-	ATP, Albumin, PROD, GGT
Bone marrow	[86]	PDMS	TCs-BMSCs and OI and TMI	2D/3D static & 3D dynamic models	NMC in 3D models	-	Flow cytometry/IFSA
	[87]	PDMS	Tumor-BM SCI and TMI	3D model	NMC	-	μSCeNT and IFSA
	[88]	PDMS	Tumor- non-tumor interaction and TMI	3D model	NMC	-	IFSA
Intestine	[104]	PDMS	TMI	Spheroid	NMC	-	MTT and IFSA
Breast	[135]	PDMS	3D tumor/homotypic	3D cell culture/monoculture	Embedded collagen-gel matrix	Manually	ATP, SDISECM
	[97]	PDMS	Tumor-matrix interaction	HD and spheroid	NMC	-	MTT and new color intensity measurement

## Abbreviations

HNSC	Human non-small cell
LC	Lung cancer
HDMEC	Human dermal microvascular endothelial cells
HFFCL	Human fetal fibroblast cell line
EH	Encapsulated in hydrogel
EM	Encapsulated in matrigel
IFSA	Immunoflorescence staining assay
HDT	Hanging droptechnique
μFCCS	Microfluidic channel-based cell culture system
ccw	Co-culture with
SMA	Stromal migration assay
TSI	Tumor–stromal interaction
TCs	Tumor cell
BM	Bone marrow
SCs	Stromal cells
OI	Osteoblasts interactions
TMI	Tumor-matrix interactions
SCI	Stromal cell interaction
